# Extramedullary versus intramedullary fixation of stable trochanteric femoral fractures: a systematic review and meta-analysis

**DOI:** 10.1007/s00402-023-04902-1

**Published:** 2023-05-02

**Authors:** Miliaan L. Zeelenberg, Leendert H. T. Nugteren, A. Cornelis Plaisier, Sverre A. I. Loggers, Pieter Joosse, Dennis Den Hartog, Michael H. J. Verhofstad, Esther M. M. van Lieshout, STABLE-HIP Study Group, Taco Gosens, Johannes H. Hegeman, Suzanne Polinder, Rudolf W. Poolman, Hanna C. Willems, Rutger G. Zuurmond

**Affiliations:** 1 Trauma Research Unit, Department of Surgery, Erasmus MC, University Medical Center Rotterdam, P.O. Box 2040, 3000 CA Rotterdam, The Netherlands; 2 Department of Surgery, Noordwest Ziekenhuisgroep, Alkmaar, The Netherlands

**Keywords:** Trochanteric, Hip fracture, Intramedullary, Extramedullary, AO type 31-A1

## Abstract

**Objective:**

This systematic review and meta-analysis compared extramedullary fixation and intramedullary fixation for stable two-part trochanteric femoral fractures (AO type 31-A1) with regards to functional outcomes, complications, and surgical outcomes.

**Methods:**

Embase, Medline, Web of Science, Cochrane Central Register of Controlled Trials, and Google Scholar were searched for randomized controlled trials (RCTs) and observational studies. Effect estimates were pooled across studies using random effects models. Results were presented as weighted risk ratio (RR) or weighted mean difference (MD) with corresponding 95% confidence interval (95% CI).

**Results:**

Five RCTs (397 patients) and 14 observational studies (21,396 patients) were included. No significant differences in functional outcomes, complications, or surgical outcomes were found between extramedullary and intramedullary fixation devices, except for a difference in duration of surgery (MD 14.1 min, CI 5.76–22.33, *p* < 0.001) and intra-operative blood loss (MD 92.30 mL, CI 13.49–171.12, *p* = 0.02), favoring intramedullary fixation.

**Conclusion:**

Current literature shows no meaningful differences in complications, surgical, or functional outcomes between extramedullary and intramedullary fixation of stable two-part trochanteric femoral fractures. Both treatment options result in good outcomes. This study implicates that, costs should be taken into account when considering implants or comparing fixation methods in future research.

**Supplementary Information:**

The online version contains supplementary material available at 10.1007/s00402-023-04902-1.

## Introduction

Proximal femoral fractures are one of the most common types of fracture in the elderly population worldwide with a global incidence of over 182.5 per 100,000 person-years [1–3]. The total annual medical costs associated with proximal femoral fractures are over $50,000 per patient in the U.S or over €20,000 in the Netherlands [4, 5]. Due to an aging population and increased life expectancy worldwide, the burden of these fractures on health care systems is increasing. About half of proximal femoral fractures are trochanteric fractures (AO type 31-A1, A2, or A3) [3]. These can be divided in stable two-part fractures (AO type 31-A1) and unstable fractures (AO type 31-A2 and A3) [6]. Of these fractures, type 31-A1 fractures make up 36% [7].

Various studies suggest that intramedullary fixation is the most appropriate approach for treatment of unstable fractures [8–10]. For stable fractures, the implant of choice remains a topic of debate. A plethora of fixation devices for both intramedullary and extramedullary fixation is available and high variability in implant preference exists between countries, hospitals, and even individual surgeons [11].

The use of intramedullary fixation devices in both stable and unstable trochanteric fractures has risen in recent years [12, 13]. Less than 2 decades ago, intramedullary fixation was discouraged as older reviews reported higher risks of revision, reoperation, and other complications [10, 14–16]. However, recent studies, that directly compared extramedullary and intramedullary fixation of stable two-part trochanteric fractures and a review of AO type A1–A3 trochanteric fractures reported none to minor differences favoring one of the two fixation types [17–21]. Due to an increased experience, development of new devices, and abolition of devices of lower quality with inferior results, the present-day intramedullary devices result in better results and are no longer inferior to extramedullary devices [22]. However, superiority over extramedullary devices has never been proven.

Therefore, current surgical guidelines, such as the Dutch Guideline for treatment of proximal femoral fractures and United Kingdom’s NICE (National Institute for Health and Care Excellence) guideline, state that due to a lack of difference in effectiveness between extramedullary and intramedullary fixation, the device with the lowest costs (i.e., a sliding hip screw) is preferred [23, 24]. The optimal and most cost-effective treatment remains a topic of debate and the steep increase in the use of intramedullary fixation may be influenced by other factors than only clinical data on complications or outcomes, such as individual preference, training, or geographical differences [12, 25].

As no extensive systematic review and meta-analysis of the literature has been conducted on treatment of, exclusively, stable two-part trochanteric fractures, the aim of this study was to compare extramedullary versus intramedullary fixation for stable two-part trochanteric femoral fractures (AO type 31-A1) only using present-day devices.

## Methods

This systematic review and meta-analysis was performed in accordance with the Preferred Reporting Items for Systematic Reviews and Meta-analysis (PRISMA) guideline [26]. A protocol was developed prior to conducting the current study.

### Search strategy and selection criteria

Embase, Pubmed/Medline, Web of Science databases, and the Cochrane Central Register of Controlled Trials (CENTRAL) were initially searched on 22 March 2021 for studies comparing extramedullary and intramedullary fixation of AO type trochanteric fractures. The initial search was updated on 26 September 2022. Online Resource 1 shows the search string used and the search results. After deduplication, two reviewers (LHTN and ACP) independently screened titles and abstracts for eligibility for inclusion. Any disagreement was resolved by consensus. The same two reviewers subsequently conducted the full-text screening of eligible articles.

Studies were included when they presented data (a) published after 1990 of (b) acute (c) AO-OTA 31-A1 trochanteric fractures, (d) comparing intramedullary and extramedullary fixation (e) in patients > 50 years, (f) using currently available devices (an overview of included devices per study is provided in Online Resource 2). Studies were excluded when they (a) presented no original data, (b) did not mention relevant outcomes (e.g. only incidence of fixation with specific devices), (c) were biomechanical, in vitro or cadaveric studies, (d) pathological fractures, (e) bilateral fractures, (f) periprosthetic fractures, (g) were case reports, or (h) did not make distinction between type of fracture or treatment.

### Data extraction

All baseline characteristics were independently extracted by two reviewers (LHTN and ACP) using a predefined data extraction sheet and included: first author, publication year, setting, study design, study period, follow-up time, total study population, and study population with type 31-A1 fracture. In addition, patient characteristics were collected including age, sex, implant type, and number of patients treated using extramedullary or intramedullary fixation.

Data were collected for the following outcome measures: functional outcomes: Harris hip score, pain, parker mobility score, and walking ability (cases of unassisted/good walking ability were compared with assisted or no walking ability). Complications: reoperation, non-union, cut-out, peri-implant fracture, conversion to prosthesis, implant/fixation failure, superficial and deep infection, malunion, limb-length discrepancy, heterotopic ossification, osteolysis in fixed implants, and mortality. Surgical outcomes/operation characteristics: operation time, blood loss, bone-healing time, quality of reduction, hospital stay, fluoroscopy time, and costs/cost-effectiveness. All outcomes were included as defined by individual presenting articles.

If case data were not described sufficiently in the full-text or supplementary materials, the authors were contacted by e-mail once. If this approach was unsuccessful, missing data were imputed based on the average standard deviation for the total included population across all studies with no missing data and adjusted for population size. This was done for the standard deviations of Harris hip score in Sevinc et al*.*[27]*,* the standard deviations of 1-year pain scores by Matre et al*.* [28]*,* and standard deviations for bone-healing time by Cho et al*.* [29].

### Quality assessment

Two reviewers (LHTN and ACP) independently assessed the methodological quality of all included studies using the risk of bias 2 (RoB 2) tool for assessing risk of bias in randomized trials and MINORS, a methodological index for non-randomized studies [30, 31]. Disagreement was resolved by consensus. The RoB 2 is structured into a fixed set of domains of bias, trial design, conduct, and reporting. Within each domain, a series of signaling questions indicate features that are relevant to risk of bias. A proposed judgement about the risk of bias for each domain is generated by an algorithm, based on the signaling questions. Judgement can be 'Low' or 'High' risk of bias, or can express 'Some concerns’. The MINORS ranges from 0 (poor quality) to 24 (high quality).

### Statistical analysis

Data were analyzed using Review Manager (Revman, version 5.3.5). Input in the analysis was sample size, mean, and standard deviation (SD) for continuous outcomes and sample size and number of cases with the specific outcome for binary outcomes. Random effects models were used for measuring treatment effects because of the expected heterogeneity due the inclusion of both RCT and observational studies and comparison of multiple types of devices in different countries and clinical settings. No (age-)adjusted analyses were performed, all data were analyzed as described in the original studies. Treatment effects of the binary outcome measures were pooled using the Cochran–Mantel–Haenszel statistic and are presented as risk ratio (RR) with 95% confidence intervals (CI). Treatment effects of the continuous outcome measures were pooled using the inverse variance weighting method and are presented as mean difference (MD) or standardized mean difference (SMD) with 95% confidence intervals (CI). SMD was used when included studies used different measurement scales for the same variable. All analyses were stratified on study design (randomized controlled trials (RCTs) or observational studies) and were presented as Forest plots. Differences between pooled estimates for the two types of study design were compared using the *χ*^2^-test test. Heterogeneity between studies was assessed qualitatively using the Cochran’s *Q*-test and quantified using the *I*^2^ statistic. Statistical significance was assumed using a *p* value threshold of 0.05. Publication bias was assessed by visual inspection of funnel plots for all studied variables (Online Resource 3).

## Results

### Search

**Fig. 1:**
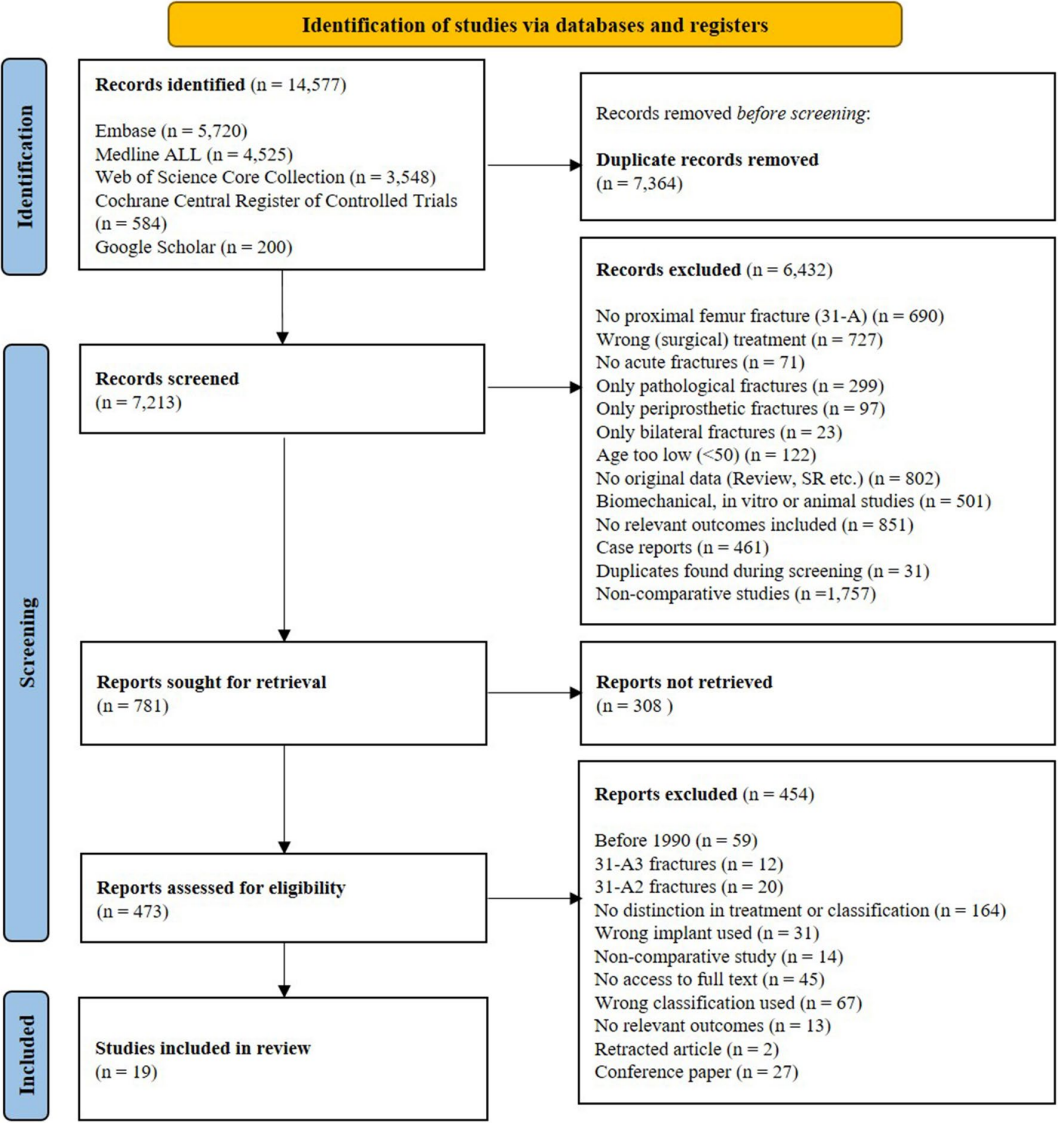
Flowchart of search results, article inclusion and exclusion

The literature search and selection of included studies is shown in Fig. 1. The primary search resulted in 14,577 records. After deduplication, 7,213 records were screened on title and abstract. Out of the 473 articles assessed for eligibility, a total of 19 studies (five RCTs [17, 32–35] and 14 observational studies [18, 19, 27–29, 36–44]) were included in the final analysis. All studies were published between 2005 and 2022.

### Baseline study characteristics

The 19 included studies (Table 1) comprised a total of 21,793 patients. The RCTs included a total of 397 patients and the observational studies a total of 21,396 patients. Extramedullary fixation was used in 15,910 patients and 5,883 patients were treated using intramedullary fixation. The follow-up ranged from 6 to 60 months. A complete overview of reported variables per study is shown in Table 2.

**Table 1:** Overview of included studies comparing intramedullary versus extramedullary fixation in AO type 31-A1 fractures

Study (publication date)	Country	Study design	Inclusion period	Total population AO type 31-A1 fracture	Extramedullary fixation				Intramedullary fixation				Follow-up (months)
					Number of patients	Device	Male (%)	Mean age (years)	Number of patients	Device	Male (%)	Mean age (years)	
RCTs													
Ovesen et al. (2006) [33]	Denmark	RCT	2001–2003	40	17	DHS	40.4^a^	78.5^a^	23	GN	79.9^a^	37.7^a^	12
Pajarinen et al. (2005) [32]	Finland	RCT	1999–2001	47	26	DHS	25.9^a^	80.3^a^	21	PFN	80.9^a^	24.9^a^	4
Parker et al. (2017) [17]	UK	RCT	2002–2009	170	83	SHS	23.2^a^	82.1^a^	87	TPF + TPFT	82.2^a^	22.4^a^	12
Tao et al. (2013) [35]	China	RCT	2010–2011	19	9	Reverse LISS	60.0	80.3	10	PFNA	80.1	22.2	12
Zou et al. (2009) [34]	China	RCT	2006–2007	121	63	DHS	24	65	58	PFNA	65	21	12
Observational studies													
Alessio-Mazzola et al*.* (2022) [41]	Italy	Retrospective cohort	2015–2019	85	44	DHS	20.5	83.7	41	PFN	14.6	86.6	41.6^c^
Andruszkow et al. (2012) [36]	Germany	Retrospective cohort	2007–2010	106	101	DHS	29.8^a,b^	80.8^a,b^	5	GN	80.8^a,b^	29.8^a,b^	N.D
Cho et al*. (*2016) [29]	South Korea	Retrospective cohort	2004–2014	194	113	DHS	38.1	84.2	81	PFNA	81	32.1	25
Crespo et al. (2012) [37]	Spain	Retrospective cohort with follow-up	2004–2009	179	125	PCCP	26.7^a^	82.5^a^	54	GN	83.1^a^	18.4^a^	12
Grønhaug et al*.* (2022) [42]	Norway	Prospective cohort	2013–2019	6,841	4,811	SHS	32	83.0	2,030	IMN	69	83.0	36
Matre et al. (2013) [28]	Norway	Retrospective cohort	2005–2010	7,643	6,355	DHS + ACHS	29	82	1,288	GN + PFNA + TITAN	82	27	22
Mohan et al. (2019) [18]	India	Retrospective cohort	2017–2018	53	23	DHS	67	45	30	PFN	60	60	6
Pehlivanoğlu et al*.* (2021) [43]	Turkey	Retrospective cohort	2005–2013	122	65	DHS	47.7	72	57	PFN	38.8	74.7	DHS 54.7^c^ PFN 44.2^c^
Pyrhönen et al*.* (2022) [44]	Sweden	Retrospective cohort	2012–2018	5,460	3,733	SHS	36.1	84	1,727	IMN	31.0	85	60
Sevinç et al. (2020) [27]	Turkey	Prospective cohort	N.D	104	48	DHS	59.1^a^	77.1^a^	56	PFNA	78.9^a^	48.2^a^	12
Talmaç et al. (2019) [19]	Turkey	Retrospective cohort	2011–2016	203	68 + 62	DHS + PCCP	52.5	70.9	73	PFNA	73.9	44.4	12
Tian et al. (2010) [39]	China	Retrospective cohort	2013–2017	58	20	DHS	40.0	70.4	38	PFNA	73.1	68.4	24
Van der Sijp et al*.* (2021) [40]	The Netherlands	Prospective cohort	2016–2018	126	32	DHS	28.1	81.3	94	PFNA	80.9	24.2	12
Yu et al. (2016) [38]	China	Retrospective cohort	2005–2015	222	112	DHS	50.9	73.1	110	PFNA	72.0	46.3	48–60

*ACHS* AMBI/CLASSIC hip screw system, *DHS* dynamic hip screw, *EM* extramedullary fixation, *GN* gamma nail, *IM* intramedullary fixation, *ND* not described, *PCCP* percutaneous compression plating, *PFN* proximal femoral nail, *PFNA* proximal femoral nail antirotation, *Reverse LISS* reverse less invasive stabilization system, *SHS* sliding hip screw, *TITAN* Trigen intertan trochanteric antegrade nail, *TPF* Targon proximal femoral nail, *TPFT* Targon proximal femoral telescrew nail

^a^Value for total study population including AO type 31-A2 and/or A3 fractures

^b^Value for individual groups not specified in study

^c^Mean follow-up, minimum of 12 months

**Table 2:** Outcomes measured reported in the included studies

Outcome	Corresponding figure	Alessio-Mazzola et al*.* [41]	Andruszkow et al*.* [36]	Cho et al. [29]	Crespo et al. [37]	Grønahug et al*.* [42]	Matre et al. [28]	Mohan et al. [18]	Ovesen et al. [33]	Pajarinen et al*.* [32]	Parker et al*.* [17]	Pehlivanoğlu et al*.* [43]	Pyrhönen et al*.* [44]	Sevinç et al*.* [27]	Talmaç et al*.* [19]	Tao et al*.* [35]	Tian et al*.* [39]	Van der Sijp et al. [40]	Yu et al. [38]	Zou et al*.* [34]
Harris hip score	2	** + **	**−**	**−**	**−**	**−**	**−**	** + **	**−**	**−**	**−**	**−**	**−**	** + **	** + **	** + **	**−**	** + **	** + **	**−**
Pain score	3	**−**	**−**	**−**	**−**	** + **	** + **	**−**	**−**	** + **	**−**	**−**	**−**	**−**	**−**	**−**	**−**	** + **	**−**	**−**
Parker mobility score	OR4-1	** + **	**−**	**−**	**−**	**−**	**−**	** + **	**−**	**−**	**−**	**−**	**−**	**−**	**−**	**−**	**−**	**−**	**−**	**−**
Post-operative walking ability	OR4-2	**−**	**−**	**−**	**−**	**−**	**−**	**−**	**−**	**−**	**−**	**−**	**−**	**−**	**−**	** + **	** + **	**−**	**−**	**−**
Reoperation	4	** + **	**−**	**−**	**−**	** + **	** + **	**−**	** + **	**−**	** + **	**−**	** + **	**−**	** + **	**−**	** + **	** + **	** + **	** + **
Non-union	5	** + **	**−**	** + **	**−**	**−**	** + **	** + **	**−**	**−**	** + **	**−**	**−**	**−**	** + **	**−**	** + **	**−**	** + **	** + **
Cut-out/protrusion	6	**−**	** + **	** + **	** + **	**−**	** + **	** + **	** + **	** + **	** + **	**−**	**−**	**−**	** + **	**−**	** + **	**−**	** + **	** + **
Peri-implant fracture	7	**−**	**−**	**−**	**−**	**−**	** + **	**−**	**−**	**−**	** + **	**−**	**−**	**−**	** + **	**−**	**−**	**−**	** + **	** + **
Conversion to prosthesis	8	** + **	**−**	** + **	**−**	** + **	** + **	**−**	** + **	**−**	** + **	**−**	** + **	**−**	**−**	**−**	** + **	**−**	**−**	**−**
Implant/fixation failure	9	** + **	**−**	** + **	**−**	**−**	** + **	**−**	**−**	**−**	**−**	**−**	**−**	**−**	** + **	**−**	** + **	** + **	** + **	** + **
Deep infections	OR4-3	** + **	**−**	** + **	**−**	**−**	**−**	** + **	** + **	** + **	**−**	**−**	**−**	**−**	**−**	**−**	**−**	**−**	** + **	** + **
Superficial infections	OR4-4	** + **	**−**	** + **	**−**	**−**	**−**	** + **	**−**	** + **	**−**	** + **	**−**	**−**	** + **	**−**	** + **	**−**	** + **	** + **
Mal-union	OR4-5	** + **	**−**	**−**	**−**	**−**	**−**	**−**	**−**	**−**	**−**	**−**	**−**	**−**	** + **	**−**	**−**	**−**	** + **	**−**
Limb-length discrepancy	OR4-6	**−**	**−**	** + **	**−**	**−**	**−**	**−**	**−**	**−**	**−**	**−**	**−**	**−**	** + **	**−**	**−**	**−**	** + **	**−**
Heterotopic ossification	OR4-7	** + **	**−**	**−**	**−**	**−**	**−**	**−**	**−**	**−**	**−**	**−**	**−**	**−**	** + **	**−**	**−**	**−**	** + **	**−**
Osteolysis	OR4-8	**−**	**−**	**−**	**−**	**−**	**−**	**−**	**−**	**−**	**−**	**−**	**−**	**−**	** + **	**−**	**−**	**−**	** + **	**−**
Mortality	OR4-9	**−**	**−**	**−**	**−**	**−**	**−**	**−**	**−**	**−**	**−**	**−**	**−**	**−**	** + **	**−**	**−**	** + **	**−**	**−**
Surgery duration	10	** + **	**−**	** + **	**−**	**−**	** + **	** + **	**−**	**−**	**−**	** + **	**−**	**−**	** + **	** + **	** + **	** + **	**−**	**−**
Blood loss	11	**−**	**−**	** + **	**−**	**−**	**−**	** + **	**−**	**−**	**−**	** + **	**−**	**−**	** + **	** + **	** + **	** + **	**−**	**−**
Bone healing time	OR4-10	**−**	**−**	** + **	**−**	**−**	**−**	**−**	**−**	**−**	**−**	**−**	**−**	**−**	** + **	** + **	** + **	**−**	** + **	**−**
Quality of reduction	OR4-11	** + **	**−**	**−**	**−**	**−**	**−**	**−**	**−**	**−**	**−**	**−**	**−**	**−**	** + **	** + **	**−**	** + **	** + **	**−**
Hospital stay	OR4-12	** + **	**−**	**−**	**−**	**−**	**−**	**−**	**−**	**−**	**−**	** + **	**−**	**−**	** + **	** + **	** + **	**−**	**−**	**−**
Fluoroscopy time	OR4-13	** + **	**−**	**−**	**−**	**−**	**−**	**−**	**−**	**−**	**−**	**−**	**−**	**−**	** + **	** + **	**−**	**−**	**−**	**−**

** + **outcome measure reported in study,** − **outcome measure not reported in study, *OR4* online resource 4

### Quality assessment

The details and distribution of the quality assessment using the RoB 2 for RCTs and MINORS for observational studies are described in Tables 3 and 4. The overall bias assessment in the RoB 2 ranged from high overall risk of bias in Pajarinen et al*.* [32] to low overall risk of bias in Tao et al*.* [35] and Ovesen et al*.* [33]. The mean score for the MINORS was 18 (SD 2.5) and ranged from 15 (moderate quality) to 22 (high quality).

**Table 3:**
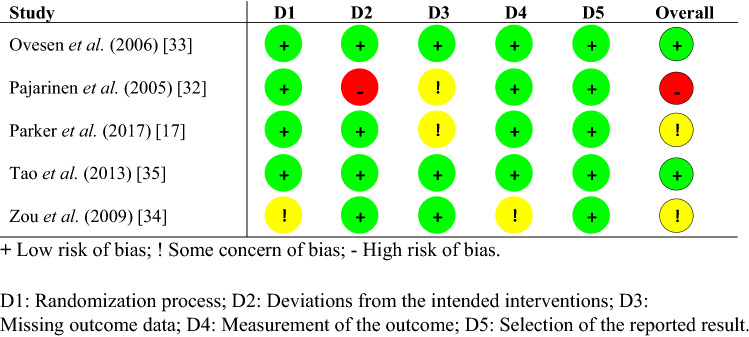
Quality assessment of included RCTs using the RoB 2 criteria

** + **low risk of bias, ! some concern of bias, − high risk of bias, *D1* randomization process, *D2* deviations from the intended interventions, *D3* missing outcome data, *D4* measurement of the outcome, *D5* selection of the reported result

**Table 4:** Quality assessment of included observational studies using the MINORS criteria

Study	Aim	Inclusion	Collection	Endpoints	Assessment	Follow-up period	Loss of follow-up	Study size calculation	Control group	Contemporary group	Baseline equivalence	Statistics	Total
Alessio-Mazzola et al*.* [41]	2	2	2	1	1	2	1	1	2	2	2	2	20
Andruszkow et al*.* [36]	2	2	0	2	0	1	2	0	1	2	1	2	15
Crespo et al*.* [37]	1	2	0	2	0	2	1	0	2	2	1	2	15
Grønhaug et al*.* [42]	2	2	2	2	0	2	1	0	2	2	2	2	19
Sevinç et al*.* [27]	2	0	2	2	0	2	2	0	2	0	1	2	15
Cho et al*.* [29]	2	2	2	2	0	2	2	0	2	2	2	2	20
Matre et al*.* [28]	2	2	1	2	0	2	1	2	2	2	2	2	20
Mohan et al*.* [18]	2	1	0	2	0	1	2	0	2	2	1	2	15
Pehlivanoğlu et al*.* [43]	2	2	2	1	0	2	0	0	2	2	1	1	15
Pyrhönen et al*.* [44]	2	2	2	2	0	2	2	0	2	2	2	2	20
Talmaç et al*.* [19]	2	2	2	2	0	2	2	0	2	2	1	2	19
Tian et al*.* [39]	2	2	0	2	0	2	2	0	2	2	1	2	17
Van der Sijp et al*.* [40]	2	2	2	2	2	2	1	2	2	2	1	2	22
Yu et al*.* [38]	1	2	1	2	0	2	2	0	2	2	2	2	18

Total scores can range from 0 up to 24, with lower scores indicating higher risk of bias

0 not reported, 1 reported but inadequate, 2 reported and adequate

A summary of all study results and meta-analyses is provided in Table 5. The most (clinically) relevant and statistically significant outcome measures are described below. Forrest plots for additional outcome measures can be found in Online Resource 4 (OR4).

**Table 5:** Summary of results of meta-analyses of extra- versus intramedullary fixation of A1 trochanteric hip fractures

Outcome measure	Pooled value	RCTs			Observational			EM fixation		IM fixation		Combined studies		
		Studies	*n*	Pooled value (95% CI)	Studies	*n*	Pooled value (95% CI)	*n*	Mean/cases	*n*	Mean/cases	Pooled value (95% CI)	*I*^2^	*p* value
Harris hip score (1 year)	MD	1	19	1.6 (− 3.9; 7.1)	5	502	1.3 (− 4.0; 6.8)	261	82.2	249	82.9	1.4 (− 3.3; 6.1)	92%	0.57
Pain score (1 year, 0–10 scale)	SMD	1	138	0.2 (− 0.2; 0.5)	3	3547	0.03 (− 0.1; 0.1)	2679	2.4	1006	2.2	0.04 (− 0.04; 0.1)	0%	0.36
Parker mobility score (1 year)	MD	x	x	x	2	114	0.52 (− 0.1; 1.1)	54	6.9	60	6.2	0.52 (− 0.1; 1.1)	0%	0.11
Good walking ability (1 year)	RR	1	19	1.1 (0.8; 1.6)	1	57	1.1 (0.7; 1.8)	28	19 (68%)	48	28 (58%)	1.1 (0.8; 1.6)	0%	0.51
Reoperation	RR	3	304	1.1 (0.2; 8.0)	8	20,633	0.94 (0.7; 1.2)	15,389	483 (3.1%)	5548	199 (3.6%)	0.94 (0.7; 1.2)	34%	0.67
Non-union	RR	2	264	No events	7	8459	1.4 (0.6; 3.4)	6933	26 (0.4%)	1790	7 (0.4%)	1.4 (0.6; 3.4)	0%	0.45
Cut-out	RR	4	351	1.1 (0.2; 5.4)	8	8659	0.7 (0.4; 1.2)	7158	36 (0.5%)	1852	20 (1.1%)	0.7 (0.4; 1.2)	0%	0.19
Peri-implant fracture	RR	2	264	No events	3	8068	1.1 (0.2; 8.2)	6732	16 (0.2%)	1600	7 (0.4%)	1.2 (0.2; 8.2)	66%	0.89
Conversion to prosthesis	RR	2	264	No events	6	20,190	0.9 (0.7; 1.2)	15,118	269 (1.8%)	5282	105 (2.0%)	0.9 (0.7; 1.2)	15%	0.32
Implant/fixation failure	RR	1	94	2.4 (0.1; 58.3)	7	8531	1.8 (0.8; 4.5)	6858	70 (1.0%)	1767	15 (0.85%)	1.8 (0.8–4.5)	20%	0.15
Deep infection	RR	3	87	No events	6	707	2.4 (0.5; 12.3)	408	4 (1.0%)	386	1 (0.3%)	2.4 (0.5; 12.3)	0%	0.30
Superficial infection	RR	2	141	2.4 (0.1; 58.3)	6	884	1.0 (0.4; 2.8)	562	9 (1.6%)	463	6 (1.3%)	1.1 (0.4; 2.9)	0%	0.81
Malunion	RR	x	x	x	3	510	0.8 (0.3; 1.8)	286	10 (3.5%)	224	10 (4.5%)	0.8 (0.3; 1.8)	0%	0.58
Limb-length discrepancy	RR	x	x	x	3	619	3.4 (0.3; 45.0)	355	11 (3.1%)	264	1 (0.3%)	3.4 (0.3; 45.0)	42%	0.36
Heterotopic ossification	RR	x	x	x	3	510	0.76 (0.3; 2.3)	286	10 (3.5%)	224	3 (4.9%)	0.76 (0.3; 2.3)	23%	0.62
Osteolysis	RR	x	x	x	2	463	0.7 (0.05; 11.0)	242	1 (0.4%)	183	1 (0.5%)	0.7 (0.05; 11.0)	0%	0.83
1-year mortality	RR	x	x	x	2	329	1.0 (0.6; 1.6)	162	31 (19%)	167	30 (18%)	1.0 (0.6; 1.6)	0%	0.92
Operation time (min)	MD	1	19	**26 (17; 36)**	8	8370	**13 (5.4; 20)**	6704	53.5	1685	53.2	**14 (7; 21)**	96%	** < 0.001**
Blood loss (mL)	MD	1	19	23 (− 54; 100)	6	641	**103 (17; 189)**	305	351	355	204	**92 (17; 171)**	98%	**0.02**
Time to union (weeks)	MD	1	19	**3.2 (0.2; 6.2)**	4	599	− 0.4 (− 1.4; 0.7)	334	17.2	284	16.9	− 0.04 (− 1.1; 1.0)	84%	0.94
Good quality of reduction	RR	1	19	0.9 (0.7; 1.2)	4	558	1.04 (0.96; 1.1)	277	191 (71%)	300	176 (59%)	1.03 (1.0; 1.1)	0%	0.46
Hospital stay (days)	MD	1	19	2.7 (− 3.7; 9.1)	4	390	0.48 (− 0.3; 1.3)	218	7.9	191	8.0	0.5 (− 0.3; 1.3)	58%	0.19
Fluoroscopy time (s)	MD	1	19	**71 (14; 128)**	2	210	7.8 (− 2.7; 18.3)	133	63.2	96	50.4	13 (− 3.2; 30)	73%	0.12

Boldface values indicate a *p* < 0.05

*EM* extramedullary, *IM* intramedullary, *MD* mean difference, *OM* outcome measure, *RCTs* randomised controlled trials, *RR* risk ratio, *x* not reported

### Functional outcomes

#### Harris hip score (HHS)

**Fig. 2:**
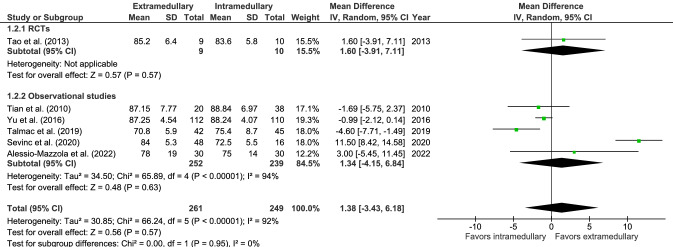
Forrest plot for Harris hip score after extramedullary versus intramedullary fixation of stable trochanteric fractures. *IV* inverse variance, *RCT* randomized controlled trial, *SD* standard deviation

The mean HHS (six studies, Fig. 2) at a minimum of 1 year after trauma was 82.2 in 261 patients for extramedullary fixation and 82.9 in 249 patients for intramedullary fixation. There was no significant difference between groups, with a considerable level of heterogeneity of effect across studies (MD 1.38, CI − 3.43 to 6.18, *I*^2^ = 92%, *p* = 0.57) [19, 27, 35, 38, 39, 41].

#### Pain

**Fig. 3:**
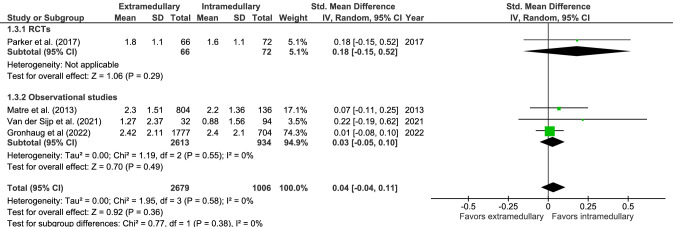
Forrest plot of 1-year pain score after extramedullary versus intramedullary fixation of stable trochanteric fractures

The mean 1-year post-operative pain score (four studies, Fig. 3) was 2.4 in 2679 patients for extramedullary fixation and 2.2 in 1006 patients for intramedullary fixation, on a 10-point scale. There was no significant difference in standardized 1-year post-operative pain between groups (SMD 0.04, CI − 0.04 to 0.11, *I*^2^ = 0%, *p* = 0.36) [17, 28, 40, 42].

#### Other functional outcomes

No significant difference between groups was found in Parker mobility score (MD 0.52, CI − 0.11 to 1.14, *I*^2^ = 0%, *p* = 0.11) (Online resource 4, Fig. 1) [18, 41] and 1-year post-operative walking ability (RR 1.11, CI 0.82–1.50, *I*^2^ = 0%, *p* = 0.97) (OR4, Fig. 2) [35, 39].

### Complications

#### Reoperation

**Fig. 4:**
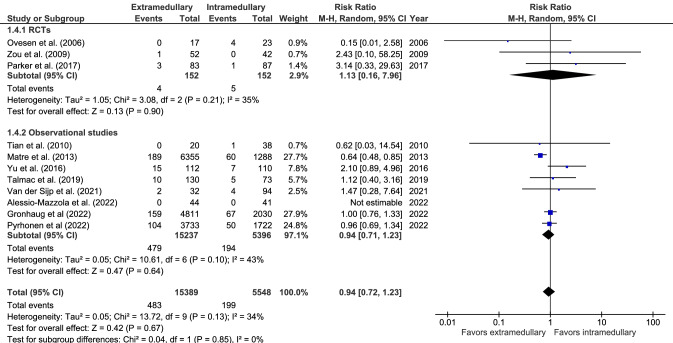
Forrest plot of reoperation rate after extramedullary versus intramedullary fixation of stable trochanteric fractures. *M–H* Mantel–Haenszel, *RCT* randomized controlled trial, *SD* standard deviation

Reoperation (11 studies, Fig. 4) was performed in 483 out of 15,389 (3.1%) in extramedullary fixation and 199 out of 5548 (3.6%) patients in intramedullary fixation. No significant difference between groups was found, with a moderate level of heterogeneity of effect across studies (RR 0.94, 0.72–1.23, *I*^2^ = 34%, *p* = 0.85) [17, 19, 28, 33, 34, 38–42, 44].

#### Nonunion

**Fig. 5:**
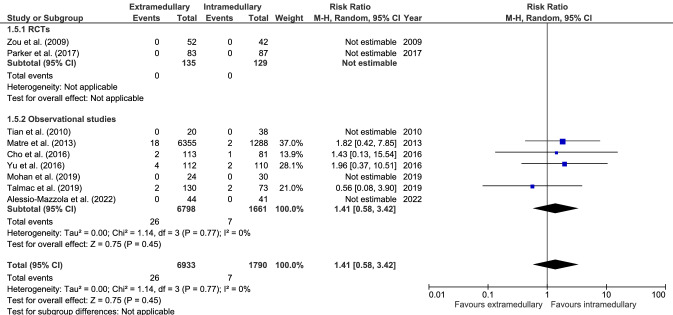
Forrest plot of non-union rate after extramedullary versus intramedullary fixation of stable trochanteric fractures

Nonunion (nine studies, Fig. 5) occurred in 26 cases out of 6933 (0.4%) in extramedullary fixation and seven cases out of 1790 (0.4%) in intramedullary fixation. There was no significant difference in non-union between groups (RR 1.41, CI 0.58–3.42, *I*^2^ = 0%, p = 0.45 [17–19, 28, 29, 34, 38, 39, 41].

#### Cut-out

**Fig. 6:**
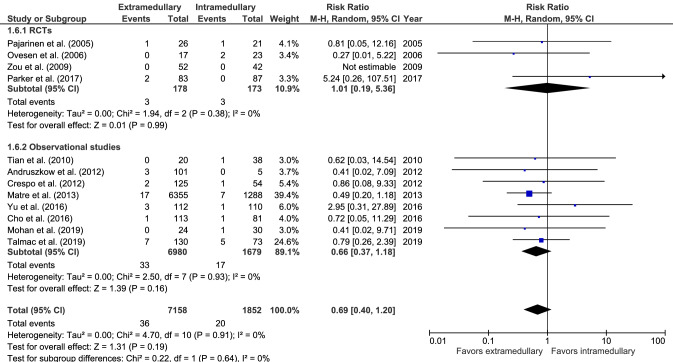
Forrest plot of cut-out rate after extramedullary versus intramedullary fixation of stable trochanteric fractures

Cut-out (12 studies, Fig. 6) occurred in 36 cases out of 7158 (0.5%) in extramedullary fixation and 20 cases out of 1852 (1.1%) in intramedullary fixation. There was no significant difference in cut-out rate between groups (RR 0.69, CI 0.40–1.20, *I*^2^ = 0%, *p* = 0.19) [17–19, 28, 29, 32–34, 36–39].

#### Peri-implant fracture

**Fig. 7:**
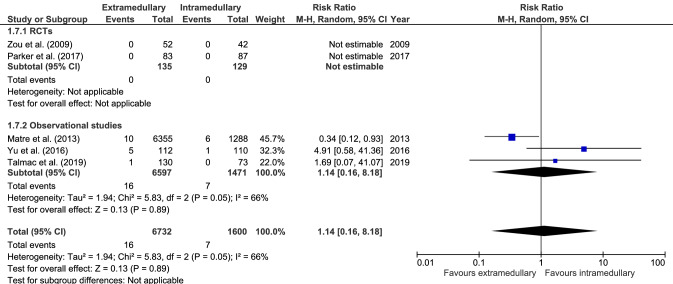
Forrest plot of peri-implant fracture rate after extramedullary versus intramedullary fixation of stable trochanteric fractures

Peri-implant fracture (five studies, Fig. 7) occurred in 16 cases out of 6732 (0.2%) in extramedullary fixation and 7 cases out of 1600 (0.4%) in intramedullary fixation. There was no significant difference in peri-implant fracture rate between fixation groups, with a moderate to substantial level of heterogeneity of effect across studies (RR 1.14, CI 0.16–8.18, *I*^2^ = 66%, p = 0.89) [17, 19, 28, 34, 38].

#### Conversion to prosthesis

**Fig. 8:**
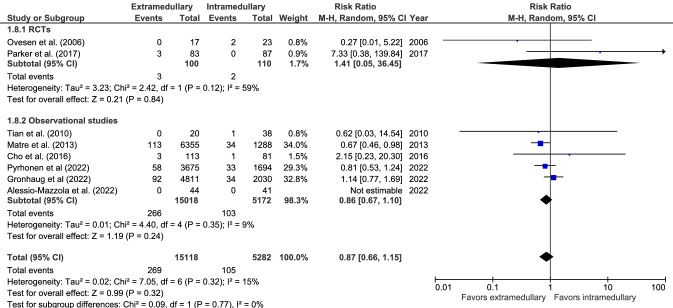
Forrest plot of conversion to prosthesis rate after extramedullary versus intramedullary fixation of stable trochanteric fractures

Conversion to prosthesis (eight studies, Fig. 8) occurred in 269 cases out 15,118 (1.8%) in extramedullary fixation and 105 cases out of 5282 (2.0%) in intramedullary fixation. There was no significant difference between groups (RR 0.87, CI 0.66–1.15, *I*^2^ = 15%, *p* = 0.32) [17, 28, 29, 33, 39, 41, 42, 44].

#### Implant/fixation failure

**Fig. 9:**
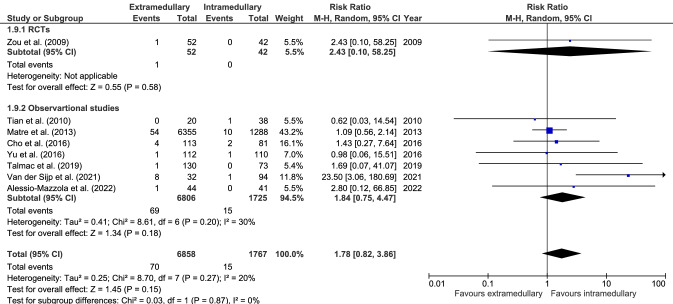
Forrest plot of implant/fixation failure rate after extramedullary versus intramedullary fixation of stable trochanteric fractures

Implant or fixation failure (eight studies, Fig. 9) was reported in 70 cases out of 6858 (1.0%) in extramedullary fixation and 15 cases out of 1767 (0.8%) in intramedullary fixation. There was no significant difference between groups (RR 1.78, CI 0.82–3.86, *I*^2^ = 20%, *p* = 0.15) [19, 28, 29, 34, 38–41].

#### Other complications

There were no significant differences between groups in deep infection (RR 2.39, CI 0.47–12.25, *I*^2^ = 0%, *p* = 0.30, OR4, Fig. 3) [18, 29, 32–34, 38, 39, 41], superficial infection (RR 1.12, CI 0.43–2.90, *I*^2^ = 0%, *p* = 0.81, OR4, Fig. 4) [19, 29, 32, 34, 38, 39, 41, 43], malunion (RR 0.78, CI 0.33–1.84, *I*^2^ = 0%, *p* = 0.58, OR4, Fig. 5) [19, 38, 41], limb-length discrepancy > 25 mm (RR 3.36, CI 0.25–45.01, *I*^2^ = 48%, *p* = 0.36, OR4, Fig. 6) [19, 29, 38], heterotopic ossification (RR 0.76, CI 0.26–2.25, *I*^2^ = 23%, *p* = 0.62, OR4, Fig. 7) [19, 38, 41], osteolysis in well-fixed implants (RR 0.74, CI 0.05–11.03, *I*^2^ = 30%, *p* = 0.83, OR4, Fig. 8) [19, 38], and 1-year mortality (RR 0.98, CI 0.61–1.56, *I*^2^ = 0%, *p* = 0.92, OR4, Fig. 9) [19, 40].

### Surgical outcomes

**Fig. 10:**
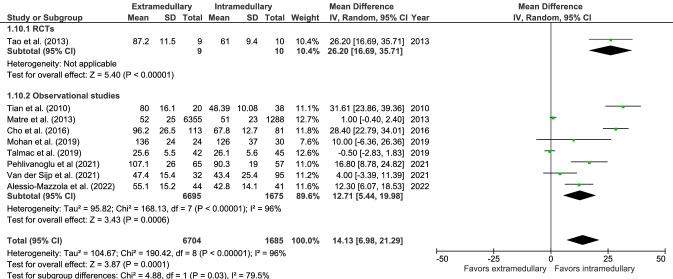
Forrest plot of mean operation time in minutes after extramedullary versus intramedullary fixation of stable trochanteric fractures

**Fig. 11:**
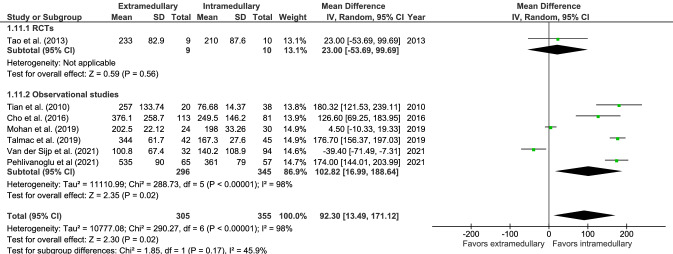
Forrest plot of mean blood loss in milliliters after extramedullary versus intramedullary fixation of stable trochanteric fractures

There was a significantly longer operation time (9 studies, Fig. 10) for extramedullary fixation, with a mean of 53.5 min in 6704 patients versus 53.2 min in 1685 patients in the intramedullary group (MD 14.1, CI 6.98–21.29, *I*^2^ = 96%, *p* < 0.001) [18, 19, 28, 29, 35, 39–41, 43]. Operative blood loss (7 studies, Fig. 11) in the extramedullary group was significantly higher as well, with a mean of 351 mL in 305 patients versus a mean of 204 mL in 355 patients in the intramedullary group (MD 92.30, CI 13.49–171.12, *I*^2^ = 98%, *p* = 0.02) [18, 19, 29, 35, 39, 40, 43].

No significant differences between the groups were found in the other surgical outcomes or operation characteristics: time to radiological union (MD − 0.04, CI − 1.10 to 1.02, *I*^2^ = 84%, *p* = 0.94, OR4, Fig. 10) [19, 29, 35, 38, 39], good quality of reduction (RR 1.03, CI 0.95–1.11, *I*^2^ = 0%, *p* = 0.46, OR4, Fig. 11) [19, 35, 38, 40, 41], hospital stay (MD 0.50, CI − 0.25 to 1.25, *I*^2^ = 48%, *p* = 0.19, Online Resource 4, Fig. 12) [19, 35, 39, 41, 43], or fluoroscopy time (MD 13.12, CI − 3.28 to 29.52, *I*^2^ = 73%, *p* = 0.12, Online Resource 4, Fig. 13) [19, 35, 41].

Only two studies reported data on hospital or surgery-related costs [41, 43]. Due to differences in included cost variables, no pooled effect could be calculated. None of the included studies reported data on cost-effectiveness.

## Discussion

This systematic review and meta-analysis compared extramedullary and intramedullary fixation for stable two-part trochanteric fractures (AO 31-A1). No differences between fixation groups were found in functional outcomes and complications. Duration of surgery and intra-operative blood loss were found to be statically different between fixation groups, in favor of intramedullary fixation, though with a high level of heterogeneity.

This is the first systematic review and meta-analysis that restricted the evaluation of treatment effect, in present-day devices, to patients with only stable two-part trochanteric fractures. It includes a substantially larger number of patients than all previous meta-analyses [9, 14, 21, 22, 45, 46]. The most recent Cochrane review, by Lewis et al. [45], on RCTs and ‘RCT-like’ cohort studies published up to July 2020 compared EM and IM fixation for a combination of AO A1-A3 fractures. In the only stratified analysis on A1 fractures, and in accordance with the current study’s data, it found no differences between fixation groups for reoperation. For all combined fractures, it found no difference in reoperation, mortality, and several functional outcomes. Contrary to this review, it did find an increased risk of many complications including non-union, both superficial and deep infection, cut-out and implant failure for extramedullary devices. Intramedullary devices were associated with an increased intra- and post-operative periprosthetic fracture rate. The differences between this Cochrane review and our meta-analysis can largely be explained by the absence of several recently published large cohort studies, its combination of both stable, and unstable fractures, and inclusion of older studies with a relatively higher rate of complications.

Another review by Wessels et al*.* [21]*,* comparing SHS vs IMN for AO A1-A3 trochanteric fractures, also found no significant differences between fixation devices in the A1 fracture subgroup. Wessels et al*.* only described a combination of major complications and, specifically, non-union. Both Lewis et al*.* and Wessels et al*.* did not include surgical outcomes or operation characteristics and also included pathological fractures.

Older reviews by Parker et al*.* [46] and Jones et al*.* [14] studied fixation of all types of trochanteric fractures by cephalomedullary nail or sliding hip screw. As opposed to the current meta-analysis and review by Wessels et al., they reported a significantly higher number of post- and intra-operative femoral fractures and higher reoperation rate for patients treated with a cephalomedullary nail. Both did not provide a stratified analysis for type of fracture. The difference between Jones et al*.*, our findings and other recent reviews is likely caused by the fact that it included older studies, from the earlier days of intramedullary fixation. Since then, device quality, design and surgical experience with intramedullary devices have greatly improved. Where older research advised against the use of intramedullary fixation, current data on clinical and functional outcomes do not warrant any statement on the preferred type of device from a clinical perspective.

### Interpretation of results

This meta-analysis found that only the duration of surgery and intra-operative blood loss to be statistically significantly in favor of intramedullary fixation. These results should be interpreted with care. The level of heterogeneity of effects across the studies was over 90% with large differences in mean operation time or blood loss (e.g. a range of 25.6–136 min for extramedullary fixation [18, 19]). This large variety may partly be explained by differences in surgery protocols, device used, experience of surgeons, the small number of inclusions, and inclusion or exclusion of anesthesia times across the included studies. The largest study by Matre et al. [28], with 7643 patients, and arguably the best sample size for this comparison, found no significant difference in operation time. The only other review reporting duration of surgery, by Parker et al*.* [46], included studies all conducted before the year 2005 and concluded that no definitive conclusion could be drawn because of limited data.

As current literature shows no differences between intra- and extramedullary fixation in functional outcomes or complications, it is tempting to conclude that surgeons could use either type of device. However, the difference in device costs should also be considered in this decision. Extramedullary fixation or more specifically dynamic/sliding hip screw is most likely the most cost-effective implant [47, 48]. Intramedullary devices can cost well over $1000 more than extramedullary options with, as demonstrated by this review, the same surgical and clinical results for stable fractures. More expensive devices, such as PFNA, should be avoided when enough surgical expertise with extramedullary devices exists. This financial argument is also used in the Dutch Guideline for treatment of proximal femoral fractures and NICE guideline, as an argument for usage of extramedullary devices when clear evidence for superiority is absent [23, 24].

Some limitations should be considered when interpreting the results of this study: First, in every meta-analysis, there is a chance of publication bias, however, the funnel plots show no clear indication for one-sided publication for the included variables. The large majority of patients (> 98%) included in this study came from observational studies resulting in a larger risk of selection bias. Many of the studied variables have a moderate-to-high level of heterogeneity between studies. Part of this could be explained by random chance and can be corrected for by using random effect models. Another part could be explained by the (small) differences in devices used, different moments in time studies were conducted, differences in study populations (e.g. age, fracture type prevalence, and inclusion of patients with concomitant injuries), and international differences between included studies. Most of the included RCTs and to a lesser degree, observational studies appear underpowered or only adequately powered for the total group of combined stable and unstable trochanteric femoral fractures. As many of the studied outcomes and complications are rare, and occur at rates of 1% or lower, studies are often not adequately powered to study these outcomes for a restricted group of stable fractures. A combination of heterogeneity and low power could conceal treatment effects that now seem insignificant. Although the clinical relevance of these effects may be questionable. Larger and adequately powered RCTs or high-quality prospective observational studies comparing extramedullary and intramedullary fixation in, specifically, stable trochanteric femoral fractures are needed to provide a definitive answer to the question of superiority for treatment for stable trochanteric fractures. Considering the low frequency of complications and sparsity of data on functional outcome and patient reported outcomes, future research should focus on both functional and quality of life data, and provide a large scale cost-effectiveness analysis.

## Conclusion

There are no meaningful differences in complications, surgical-, or functional outcomes between intramedullary and extramedullary fixation of stable two-part trochanteric femoral (AO type 31-A1) fractures. Both treatment options result in good outcomes and few complications. As outcomes do not differ, costs should be taken into account when considering devices. Therefore, the use of extramedullary devices should be advised when enough surgical expertise with these devices is available. Future research should focus on (functional) outcomes for individual fracture AO type A1–A3 subgroups, and cost-effectivity of treatment and (medical) decision making for both techniques.

## Supplementary Information

Below is the link to the electronic supplementary material.

Supplementary file1 (DOCX 17 KB)

Supplementary file2 (DOCX 16 KB)

Supplementary file3 (DOCX 116 KB)

Supplementary file4 (DOCX 205 KB)

## Data Availability

All data used in this meta-analysis were published in previous literature. Specific information, on the data used or the analysis performed, is available upon request from the corresponding author.
