# Supplementary material for: Extramedullary versus intramedullary fixation of stable trochanteric femoral fractures: a systematic review and meta-analysis
Source: Arch Orthop Trauma Surg. 2023 May 2;143(8):5065–83. doi: 10.1007/s00402-023-04902-1 (PMC10374813; doi:10.1007/s00402-023-04902-1)
Supplement: Supplementary file 2 — Supplementary file2 (DOCX 16 KB) [file 402_2023_4902_MOESM2_ESM.docx]

**Online resource 2: Intramedullary and extramedullary devices**

**Table 1: Specific devices used in included studies as mentioned by authors**

|  | **Intramedullary device** | **Extramedullary device** |
| --- | --- | --- |
| **RCTs** | | |
| Ovesen *et al*. (2006) | Trochanteric gamma nail (GN)^1^, second generation | Dynamic hip screw (DHS)^2^, with or without stabilizing plate |
| Pajarinen *et al*. (2005) | Proximal femoral nail (PFN)^2^ | Dynamic hip screw (DHS)^2^ |
| Parker *et al*. (2017) | Targon proximal femoral (TPF) nail^3^ + Targon proximal femoral telescrew (TPFT) nail^3^ | Sliding hip screw (SHS), NFS |
| Tao *et al*. (2013) | Proximal femoral nail antirotation (PFNA)^2^ | Reverse less invasive stabilization system (LISS)^2^ |
| Zou *et al*. (2009) | Proximal femoral nail antirotation (PFNA)^2^ | Dynamic hip screw (DHS)^2^ |
| **Observational studies** | | |
| Alessio-Mazzola *et al.* (2022) | Intramedullary proximal femoral nail (PFN)^3^ | Dynamic hip screw (DHS)^2^ |
| Andruszkow *et al*. (2012) | Gamma 3 nail (GN)^1^ | Dynamic hip screw (DHS)^2^ |
| Cho *et al. (*2016) | Proximal femoral nail antirotation (PFNA)^2^ | Dynamic hip screw (DHS)^2^ |
| Crespo *et al*. (2012) | Short gamma nail (GN)^1^ | Percutaneous compression plate (PCCP)^5^ |
| Grønhaug *et al.* (2022) | Intramedullary nail (IMN) short or long, NFS | Sliding hip screw (SHS) with or without a trochanteric support plate (TSP), NFS |
| Matre *et al*. (2013) | Gamma 3 nail (GN)^1^, second and third generation + Proximal femoral nail antirotation (PFNA)^2^ + Trigen intertan trochanteric antegrade nail^6^ | AMBI/CLASSIC hip screw system^6^ + Dynamic hip screw (DHS)^2^, with or without compression plate |
| Mohan *et al*. (2019) | Proximal femoral nail (PFN)^2^ | Dynamic hip screw (DHS)^2^ |
| Pehlivanoglu *et al.* (2021) | Proximal femoral nail antirotation (PFNA)^2^ | Dynamic hip screw (DHS)^2^ |
| Pyrhönen et al. (2022) | Intramedullary nail (IMN), short and long, NFS | Sliding hip screw (SHS,) NFS |
| Sevinç *et al*. (2020) | Proximal femoral nail antirotation (PFNA)^2^ | Dynamic hip screw (DHS)^2^ |
| Talmaç *et al*. (2019) | Proximal femoral nail antirotation (PFNA)^2^ | Dynamic hip screw (DHS)^2^ + Percutaneous compression plate (PCCP)^5^ |
| Tian *et al*. (2010) | Proximal femoral nail antirotation (PFNA)^2^ | Dynamic hip screw (DHS)^2^ |
| Van der Sijp *et al.* (2021) | Proximal femoral nail antirotation (PFNA)^2^ | Dynamic hip screw (DHS)^2^ |
| Yu *et al*. (2016) | Proximal femoral nail antirotation (PFNA)^2^ | Dynamic hip screw (DHS)^2^ |

^1^ Stryker Howmedica, Freiburg, Germany/ Mahwha, New Jersey, USA

^2^ Depuy-Synthes, Oberdorf, Switzerland/ Westchester, PA, USA

^3^ Endovis B.A., EBA, Citieffe, Italy

^4^ B. Braun, Tuttlingen, Germany

^5^ Orthofix, Bussolengo, Italy

^6^ Smith & Nephew, London, UK

NFS, not further specified

**Extramedullary versus intramedullary fixation of stable trochanteric femoral fractures: a systematic review and meta-analysis**

Archives of Orthopaedic and Trauma Surgery

Miliaan L. Zeelenberg^1^, MD; Leendert H.T. Nugteren^1^, BSc ; A. Cornelis Plaisier^1^, BSc; Sverre A.I. Loggers^1,2^, MD; Pieter Joosse^2^, MD PhD; Dennis Den Hartog^1^, MD PhD; Michiel H.J. Verhofstad^1^, MD PhD; Esther M.M. Van Lieshout^1^, PhD MSc; STABLE-HIP Study Group*

^1^Trauma Research Unit Department of Surgery, Erasmus MC, University Medical Center Rotterdam, Rotterdam, The Netherlands

^2^Department of Surgery, Noordwest Ziekenhuisgroep, Alkmaar, The Netherlands

*Taco Gosens, MD PhD; Johannes H. Hegeman, MD PhD; Suzanne Polinder, PhD; Rudolf W. Poolman, MD PhD; Hanna C. Willems, MD PhD; Rutger G. Zuurmond, MD PhD

**Corresponding authors**

Dr. E.M.M. Van Lieshout

Trauma Research Unit Department of Surgery

Erasmus MC, University Medical Center Rotterdam

P.O. Box 2040

3000 CA Rotterdam

The Netherlands

Phone: +31.10.7031050

Mail: [e.vanlieshout@erasmusmc.nl](mailto:e.vanlieshout@erasmusmc.nl)
