# Supplementary material for: Extramedullary versus intramedullary fixation of stable trochanteric femoral fractures: a systematic review and meta-analysis
Source: Arch Orthop Trauma Surg. 2023 May 2;143(8):5065–83. doi: 10.1007/s00402-023-04902-1 (PMC10374813; doi:10.1007/s00402-023-04902-1)
Supplement: Supplementary file 3 — Supplementary file3 (DOCX 116 KB) [file 402_2023_4902_MOESM3_ESM.docx]

**Online Resource 3: Funnel Plots of included outcome measures**

| **A: Harris Hip Score** | **B: One-year pain score** |
| --- | --- |
| **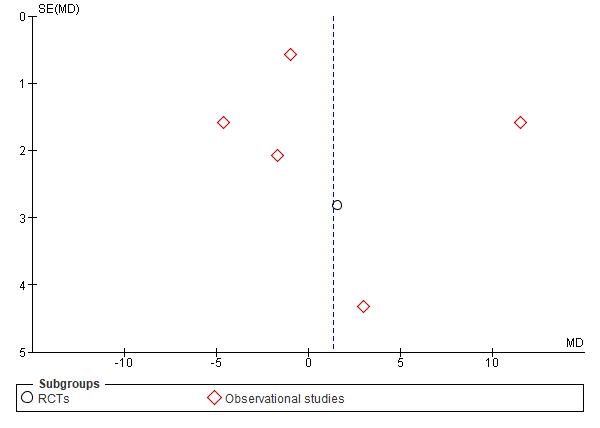** | **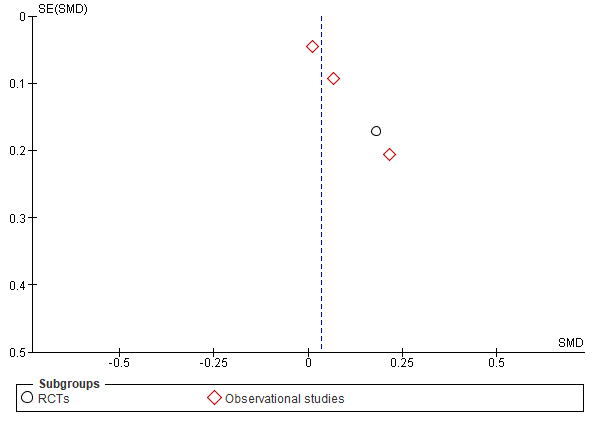** |
| **C: Parker mobility score** | **D: Good walking ability** |
| 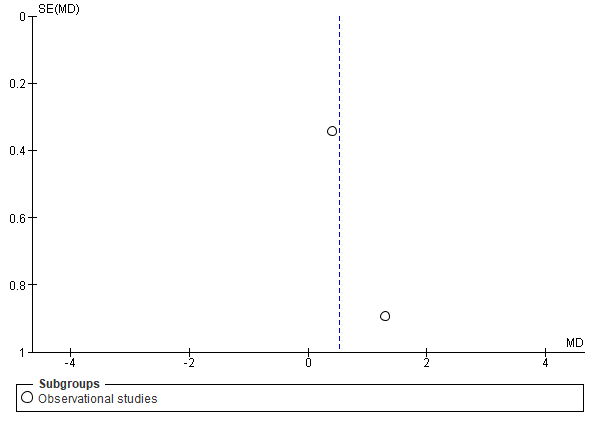 | **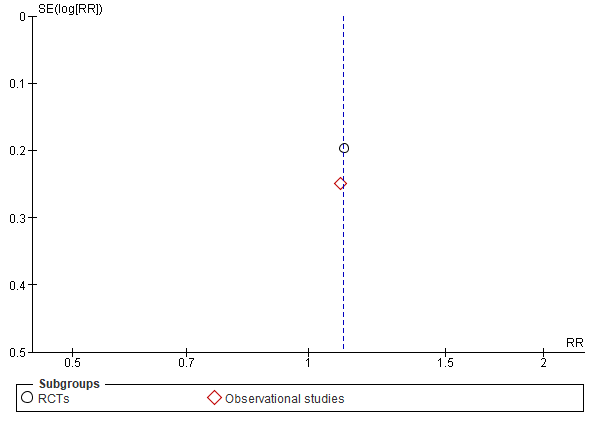** |
| **E: Reoperation** | **F: Deep infection** |
| **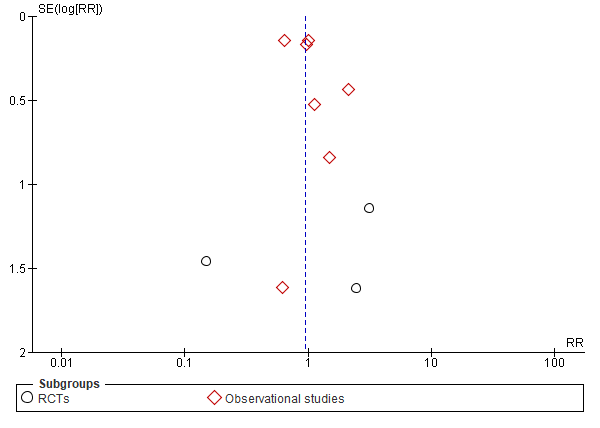** | **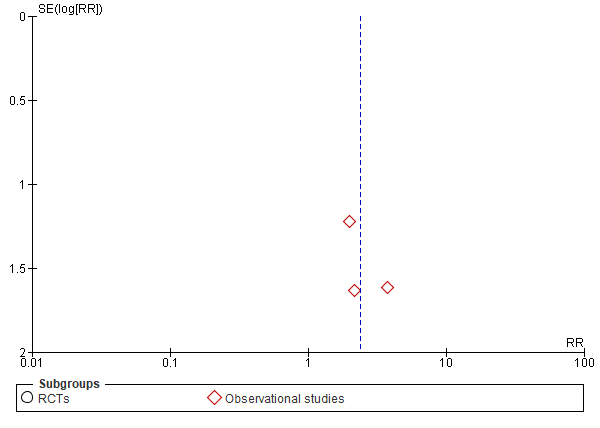** |
| **G: Superficial infection** | **H: Non-union** |
| **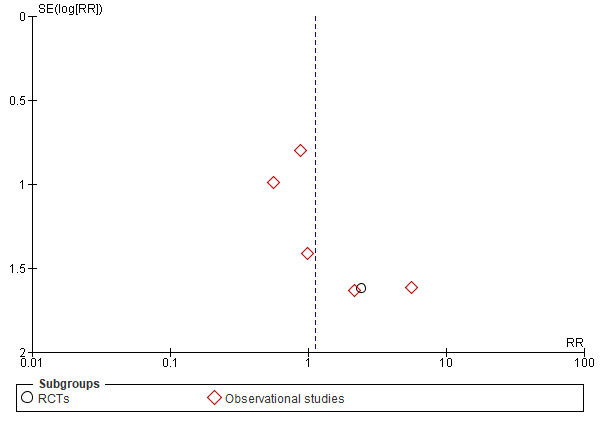** | **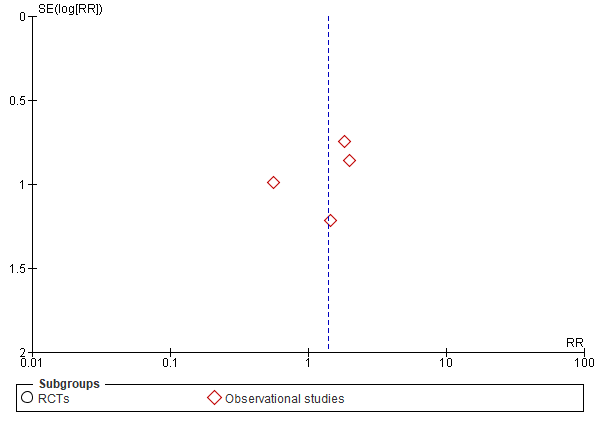** |
| **I: Malunion** | **J: Cut-out** |
| **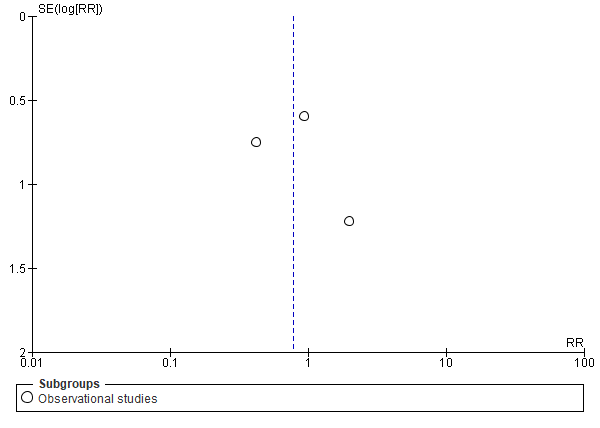** | **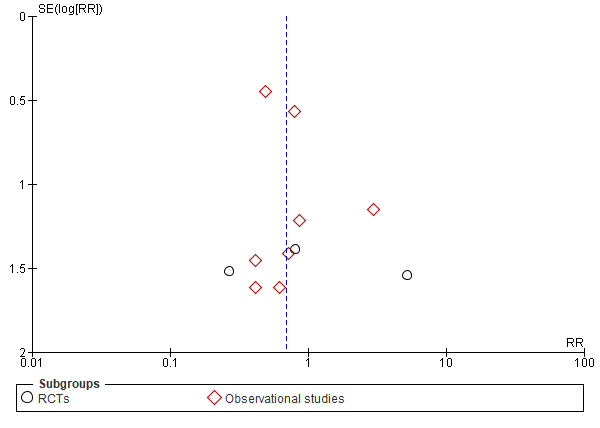** |
| **K: Peri-implant fracture** | **L: Conversion to prosthesis** |
| **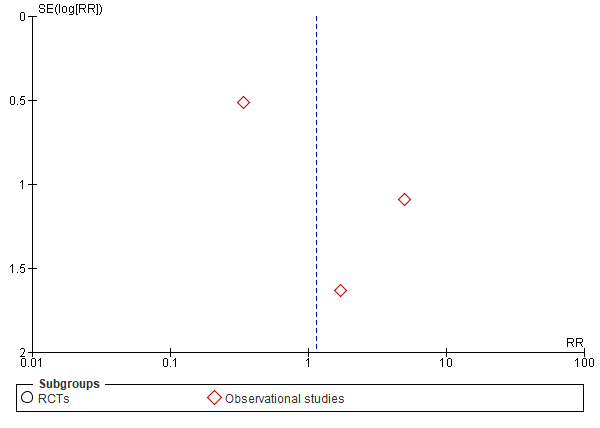** | **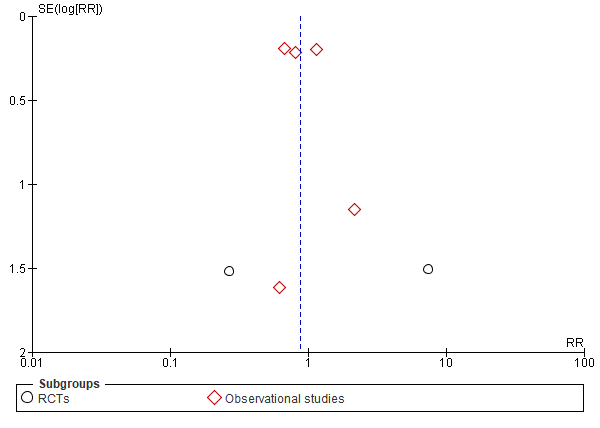** |
| **M: Implant/fixation failure** | **N: Limb-length discrepancy** |
| **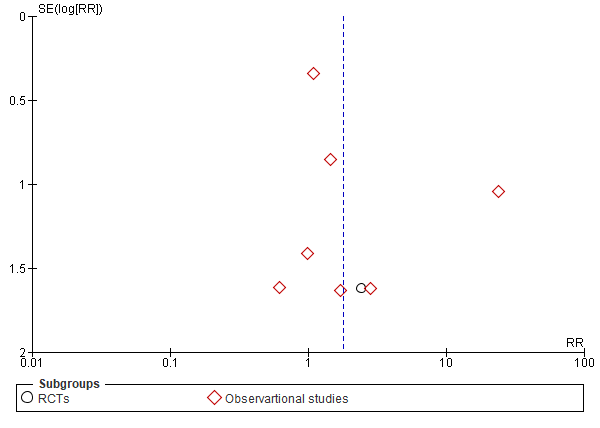** | **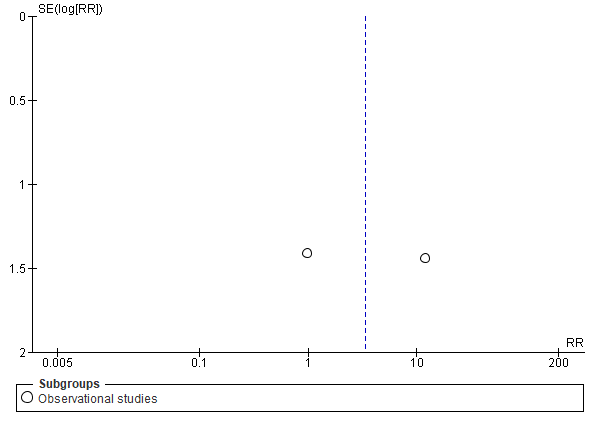** |
| **O: Heterotopic ossification** | **P: Osteolysis** |
| **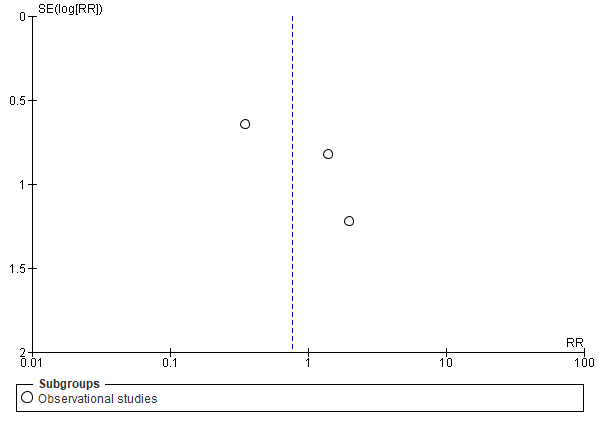** | **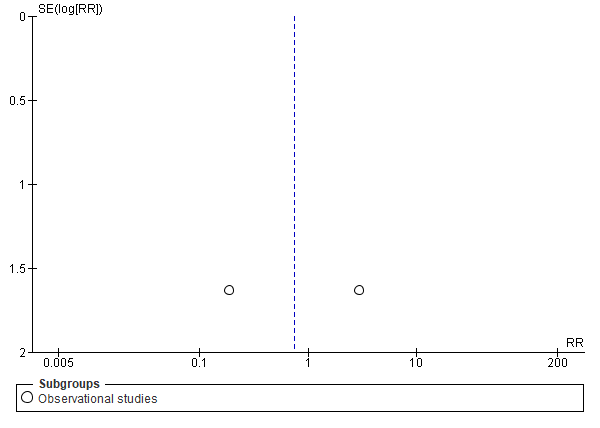** |
| **Q: Mortality** | **R: Bone healing time** |
| **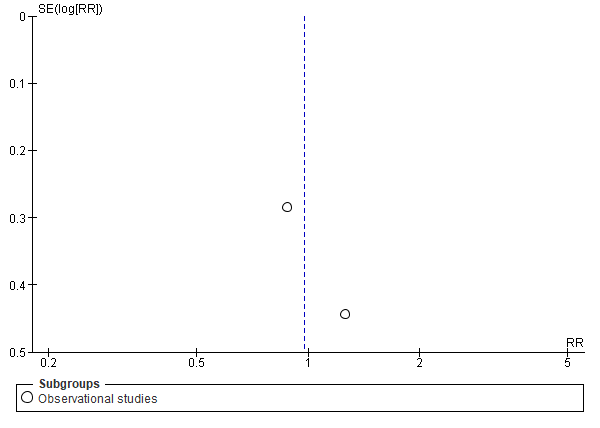** | **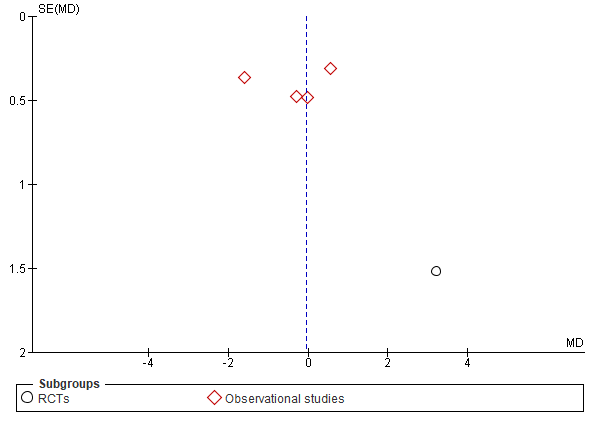** |
| **S: Good quality of reduction** | **T: Operation time** |
| **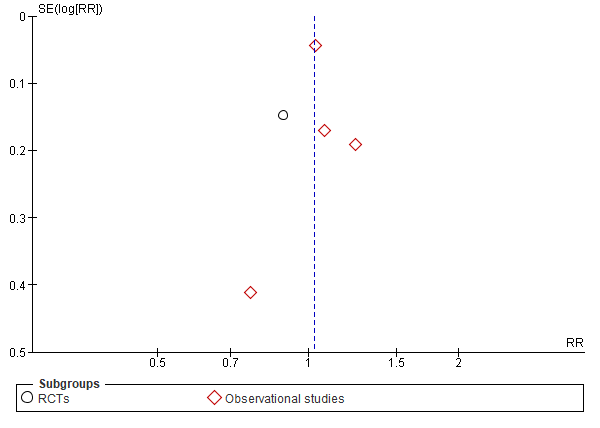** | **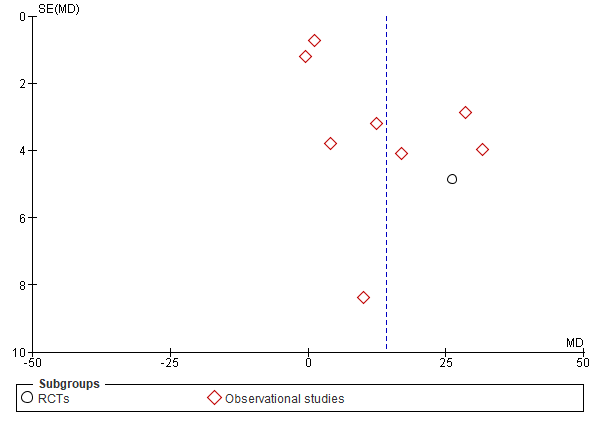** |
| **U: Hospital stay** | **V: Blood loss** |
| **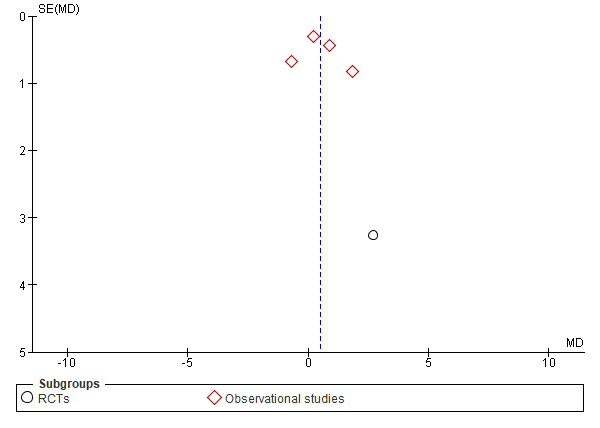** | **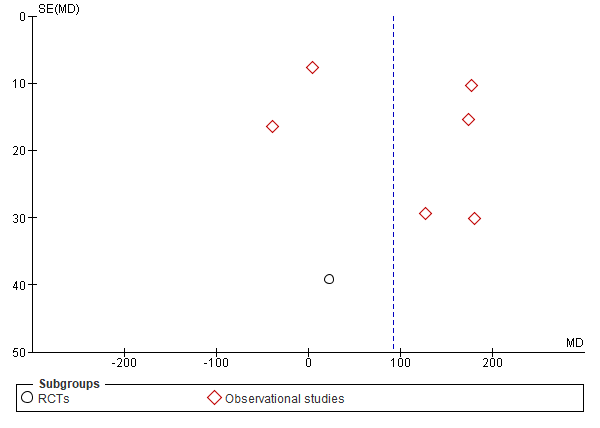** |
| **W: Fluoroscopy time** |  |
| **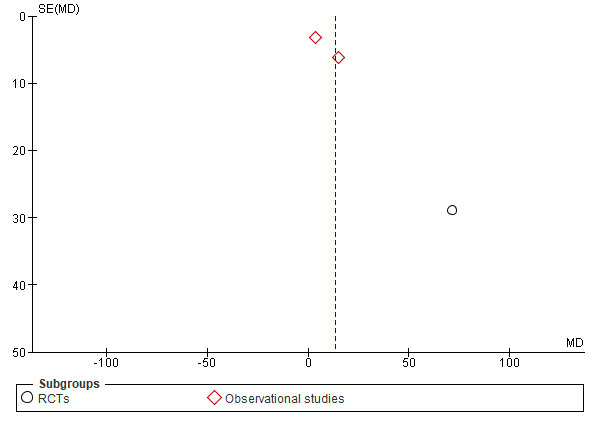** |  |

**Extramedullary versus intramedullary fixation of stable trochanteric femoral fractures: a systematic review and meta-analysis**

Archives of Orthopaedic and Trauma Surgery

Miliaan L. Zeelenberg^1^, MD; Leendert H.T. Nugteren^1^, BSc ; A. Cornelis Plaisier^1^, BSc; Sverre A.I. Loggers^1,2^, MD; Pieter Joosse^2^, MD PhD; Dennis Den Hartog^1^, MD PhD; Michiel H.J. Verhofstad^1^, MD PhD; Esther M.M. Van Lieshout^1^, PhD MSc; STABLE-HIP Study Group*

^1^Trauma Research Unit Department of Surgery, Erasmus MC, University Medical Center Rotterdam, Rotterdam, The Netherlands

^2^Department of Surgery, Noordwest Ziekenhuisgroep, Alkmaar, The Netherlands

*Taco Gosens, MD PhD; Johannes H. Hegeman, MD PhD; Suzanne Polinder, PhD; Rudolf W. Poolman, MD PhD; Hanna C. Willems, MD PhD; Rutger G. Zuurmond, MD PhD

**Corresponding authors**

Dr. E.M.M. Van Lieshout

Trauma Research Unit Department of Surgery

Erasmus MC, University Medical Center Rotterdam

P.O. Box 2040

3000 CA Rotterdam

The Netherlands

Phone: +31.10.7031050

Mail: [e.vanlieshout@erasmusmc.nl](mailto:e.vanlieshout@erasmusmc.nl)
