# Supplementary material for: Extramedullary versus intramedullary fixation of stable trochanteric femoral fractures: a systematic review and meta-analysis
Source: Arch Orthop Trauma Surg. 2023 May 2;143(8):5065–83. doi: 10.1007/s00402-023-04902-1 (PMC10374813; doi:10.1007/s00402-023-04902-1)
Supplement: Supplementary file 4 — Supplementary file4 (DOCX 205 KB) [file 402_2023_4902_MOESM4_ESM.docx]

**Online Resource 4: Additional forest plots of included variables**

**Extramedullary versus intramedullary fixation of stable trochanteric femoral fractures: a systematic review and meta-analysis**

Archives of Orthopaedic and Trauma Surgery

Miliaan L. Zeelenberg^1^, MD; Leendert H.T. Nugteren^1^, BSc ; A. Cornelis Plaisier^1^, BSc; Sverre A.I. Loggers^1,2^, MD; Pieter Joosse^2^, MD PhD; Dennis Den Hartog^1^, MD PhD; Michiel H.J. Verhofstad^1^, MD PhD; Esther M.M. Van Lieshout^1^, PhD MSc; STABLE-HIP Study Group*

^1^Trauma Research Unit Department of Surgery, Erasmus MC, University Medical Center Rotterdam, Rotterdam, The Netherlands

^2^Department of Surgery, Noordwest Ziekenhuisgroep, Alkmaar, The Netherlands

*Taco Gosens, MD PhD; Johannes H. Hegeman, MD PhD; Suzanne Polinder, PhD; Rudolf W. Poolman, MD PhD; Hanna C. Willems, MD PhD; Rutger G. Zuurmond, MD PhD

**Corresponding authors**

Dr. E.M.M. Van Lieshout

Trauma Research Unit Department of Surgery

Erasmus MC, University Medical Center Rotterdam

P.O. Box 2040

3000 CA Rotterdam

The Netherlands

Phone: +31.10.7031050

Mail: [e.vanlieshout@erasmusmc.nl](mailto:e.vanlieshout@erasmusmc.nl)

**
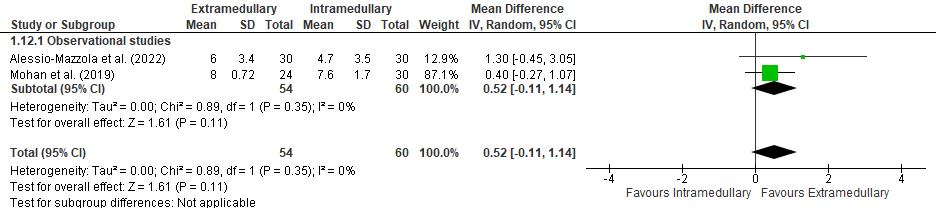
Figure 1: Forrest plot of Parker mobility score after extramedullary versus intramedullary fixation of stable trochanteric fractures**

**
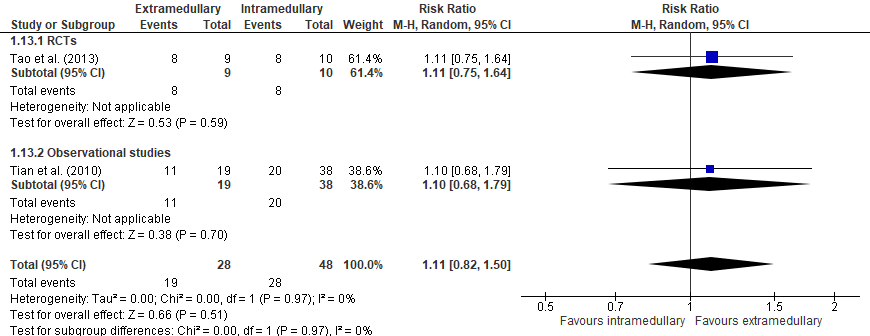
**

**Figure 2: Forrest plot of good walking ability after extramedullary versus intramedullary fixation of stable trochanteric fractures**

**
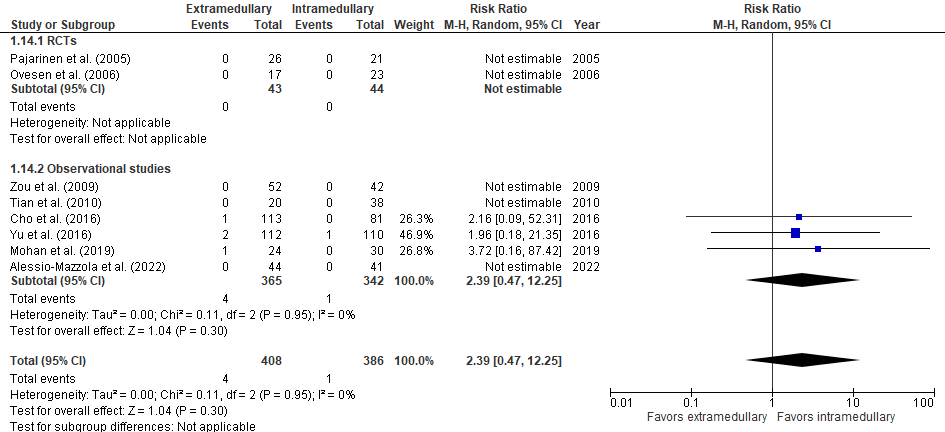
**

**Figure 3: Forrest plot of deep infection rate after extramedullary versus intramedullary fixation of stable trochanteric fractures^1^**

^1^Deep infection was defined as post-operative (wound) infection needing the use of intravenous antibiotics and/or operative intervention

**
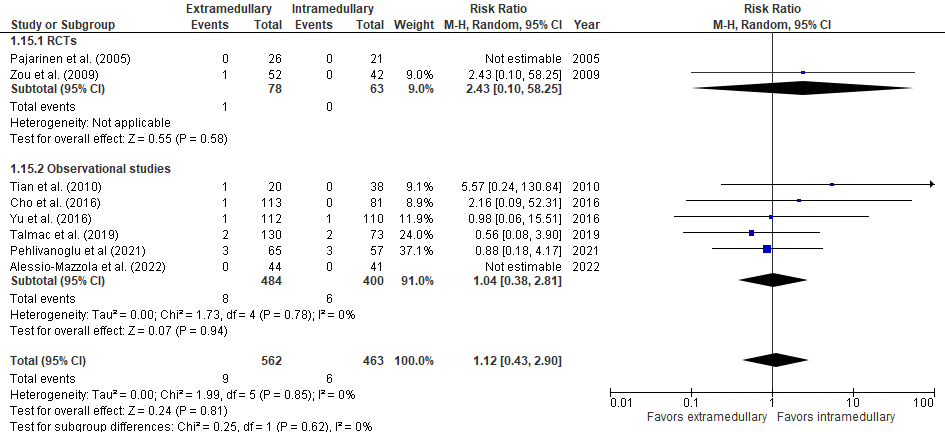
**

**Figure 4: Forrest plot of superficial infection rate after extramedullary versus intramedullary fixation of stable trochanteric fractures^1^**

^1^Superficial infection was defined as post-operative wound infection not needing intravenous antibiotics or operative intervention.


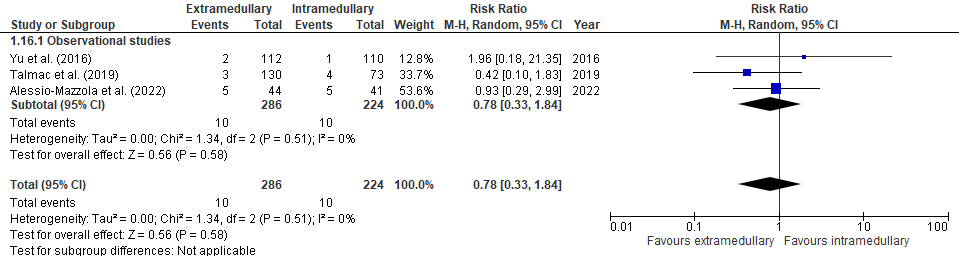


**Figure 5: Forrest plot of malunion rate after extramedullary versus intramedullary fixation of stable trochanteric fractures**

**
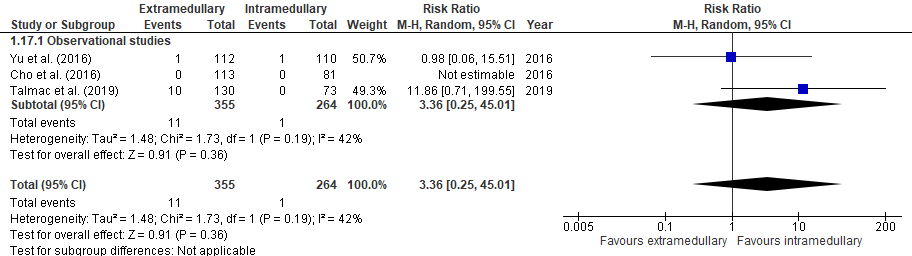
**

**Figure 6: Forrest plot of limb-length discrepancy rate after extramedullary versus intramedullary fixation of stable trochanteric fractures**

**
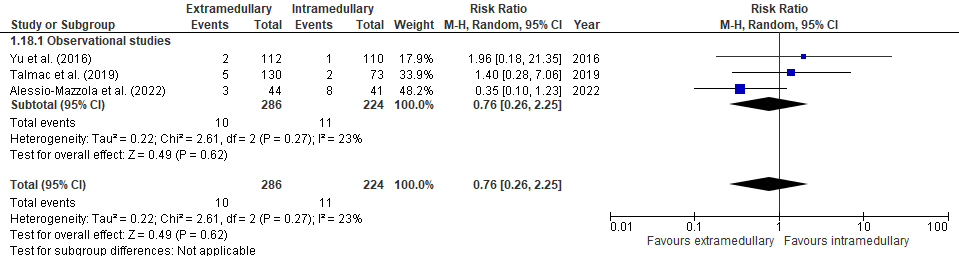
Figure 7: Forrest plot of heterotopic ossification rate after extramedullary versus intramedullary fixation of stable trochanteric fractures**

**
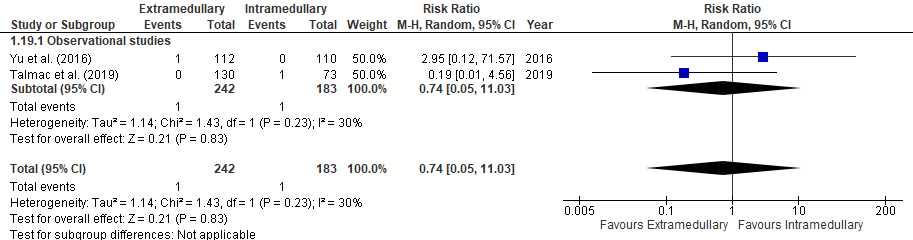
Figure 8: Forrest plot of osteolysis rate in well-fixed implants after extramedullary versus intramedullary fixation of stable trochanteric fractures**

**
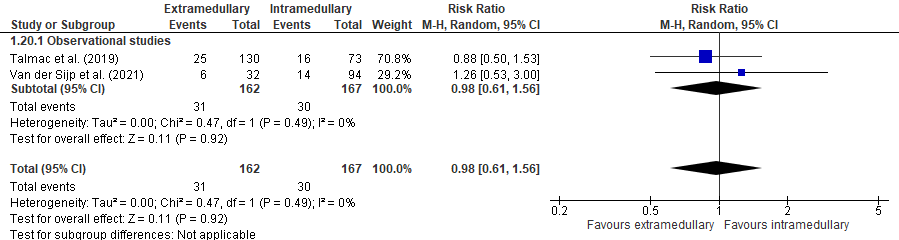
Figure 9: Forrest plot of one-year mortality rate** **after extramedullary versus intramedullary fixation of stable trochanteric fractures**

**
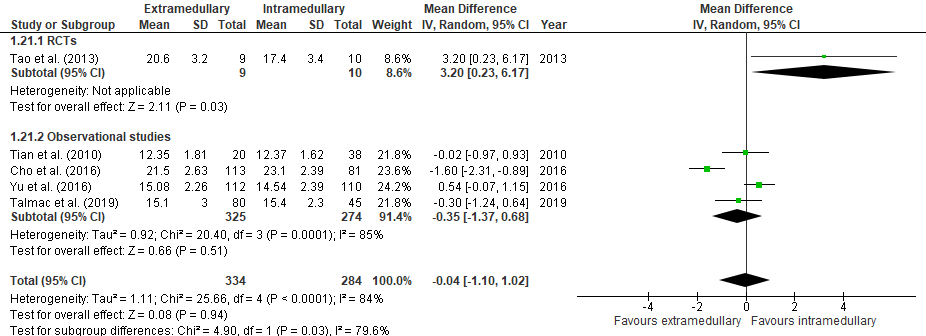
Figure 10: Forrest plot of mean bone healing time in weeks after extramedullary versus intramedullary fixation of stable trochanteric fractures**

**
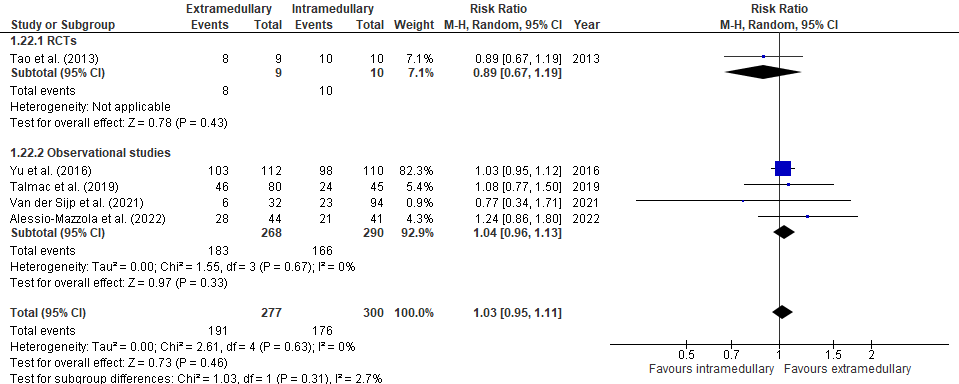
Figure 11: Forrest plot of good quality of reduction rate after extramedullary versus intramedullary fixation of stable trochanteric fractures**

**
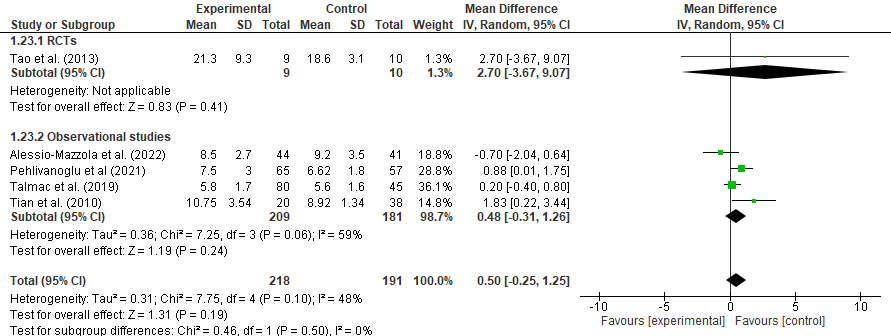
Figure 12: Forrest plot of mean hospital stay in days after extramedullary versus intramedullary fixation of stable trochanteric fractures**

**
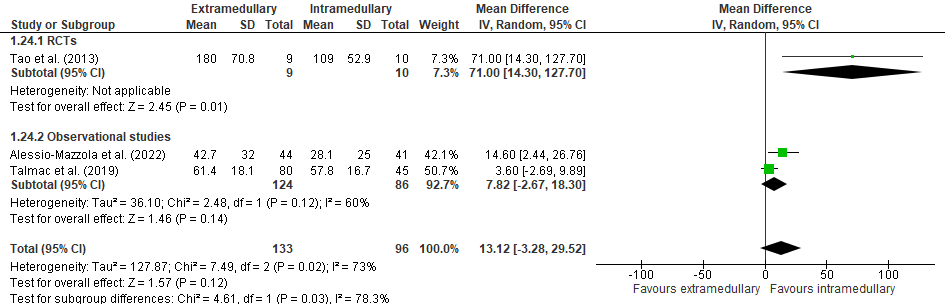
Figure 13: Forrest plot of mean fluoroscopy time in seconds after extramedullary versus intramedullary fixation of stable trochanteric fractures**
